# Fowl Cholera in Chickens: Current Trends in Diagnosis and Phenotypic Drug Resistance in Gondar City, Ethiopia

**DOI:** 10.1155/vmi/6613019

**Published:** 2024-12-05

**Authors:** Abdo Megra Geda

**Affiliations:** Department of Veterinary Pathobiology, College of Veterinary Medicine and Animal Sciences, University of Gondar, Gondar, Ethiopia

**Keywords:** chickens, fowl cholera, Gondar city, isolation, molecular detection, *Pasteurella multocida*

## Abstract

Ethiopia, with an estimated chicken population of 17 million, serves as a source of high-quality animal protein, helping to reduce malnutrition, improve nutritional status, and provide food and food products. However, Ethiopia has not fully leveraged the value of chicken production due to various bacterial diseases, with fowl cholera (FC) being the most common. Therefore, the objective of this review is to highlight the current trends in the diagnosis of FC in chickens and asses its phenotypic drug resistance patterns in Gondar City. FC is an infectious disease caused by *Pasteurella multocida* (*P. multocida*), which poses significant health and financial losses to the poultry industry. Culturally, the bacterium *P. multocida* can be isolated using bacteriological and biochemical tests from chicken infected with FC. Molecular-based techniques such as capsular and lipopolysaccharide genotyping, as well as nucleic acid amplification tests through PCR assays, are also among the best methods used to detect *P. multocida*. In conclusion, understanding the current trends in diagnosing FC and assessing its phenotyping drug resistance, which helps in choosing effective antibiotics in Gondar City, is essential. It is also important to assess the disease-associated factors that enhance the occurrence of the disease, in addition to providing the disease preventive and control measures and vaccination programs based on the diagnosis of its causative agent.

## 1. Introduction

Ethiopia is the African country estimated to have a chicken population of 57 million. Among these, approximately 78.84%, 12.05%, and 9.11% are indigenous, hybrids, and exotic breeds, respectively [1]. This large chicken population holds significant socioeconomic importance for both urban and rural communities in Ethiopia [2]. Chicken serves as a source of high-quality animal protein, which can decrease malnutrition and improve nutritional status, while also providing meat and eggs for consumption [3]. Moreover, chicken production offers a vital source of cash income for nearly 80% of the Ethiopian community through the sales of live chicken and eggs [4, 5].

Despite its potential, chicken production in Ethiopia faces significant challenges from various pathogenic viral and bacterial diseases. Among bacterial diseases, fowl cholera (FC) is a major concern and is primarily recognized as a prevalent chicken disease. It is caused by highly infectious Gram-negative bacterium *Pasteurella multocida* (*P. multocida*) [6, 7].

FC is endemic and frequently occurs sporadically, impacting the upper and lower respiratory tracts of chickens, turkeys, ducks, and geese [8, 9]. The economic impact of FC is severe, resulting in fatalities, weight loss, carcass rejection, and increased medication expenses for chicken farmers [10]. It also leads to the death of 40%–60% of newly hatched chicks before they reach maturity, resulting in high morbidity (52%) and mortality (56%) rates [9].

FC can manifest as either acute or chronic, indicated by various clinical symptoms in the affected chickens [11, 12]. For treatment, various antibiotics are commonly used; however, their widespread and prolonged use has led *P. multocida* to develop resistance to many antimicrobials [13].

Despite FC being considered one of the most prevalent and economically significant diseases [14], there is a dearth of information regarding its presence in Gondar City, where treatment is often based on tentative clinical diagnosis. There is also a notable absence of reviews and research works that show the presence and economic burden of the disease, FC, in chickens in Gondar City.

The lack of knowledge regarding these gaps hinders effective disease management and treatment strategies in the area. This highlights the necessity for a systemic monitoring and evaluation system to better understand the disease dynamics and implement appropriate control strategies based on the diagnosis of disease causative agents using phenotypic and molecular analyses, along with clinical examinations. It also suggests the need to search for alternative antibiotics based on the findings of sensitivity tests profiles. As the result, this review could provide important information for veterinarians working in veterinary clinics regarding the disease description, clinical aspects, and laboratory diagnosis of FC in chickens. In addition, it enables veterinarians to select the most effective antibiotic for the treatment of FC in Gondar City and serves as a starting point for further study. Therefore, the objective of this review is to highlight the current status of FC in Gondar City, including its diagnosing methods and phenotypic drug resistance profile.

## 2. Literature Review

### 2.1. Background of FC

In the historical background of FC, it was previously known in 1878 as fowl plague, a disease observed in poultry production in Italy and different European countries [15]. However, in 1880, the difference between these diseases (fowl plague and FC) was clarified by Luis Pasteur, a French chemist, pharmacist, and microbiologist, who played a significant role in understanding poultry diseases. FC has also been believed to have been present for a very long time, maybe for a century before being distinguished from the fowl plague. Through the study of Louis Pasteur, the bacterium, *P. multocida* causing FC was isolated in diseased chickens. FC was then first identified as a poultry disease, making it one of the earliest infectious diseases [16]. Despite being known and studied, it remains a serious and challenging poultry disease, highlighting its substantial influence on poultry health and the ongoing global efforts to combat it [14].

### 2.2. Etiology of FC

FC is frequently caused by the bacterium, *P. multocida,* capsular polysaccharide (CPS) A, and F serogroups, which together results in 80% of deaths [17]. Similarly, type D strains of FC in chicken only result in 20% mortality [18]. In addition, serotype B:3 is frequently isolated from avian disease cases presenting as sinusitis and exhibiting signs of nasal discharge, increased lacrimation, edema, and inflammation. These strains are easily destroyed by many disinfectants as well as by sunlight, heat, and drying [19].

### 2.3. Pathogenicity of *P. multocida*

Although the bacterium *P. multocida* usually exists as a commensal organism in the respiratory tracts of various avian species [20], its pathogenicity-related conditions in chickens are subjected to stress, overcrowding, poor health, or adverse environmental factors, which enhance the bacterium's ability to enter or infect the chicken host and develop the disease [21]. In the same manner, the pathogenicity of *P. multocida* also involves specific virulence genes that it encodes, particularly the *hexA* and *hyaD/hyaC* genes, which play a crucial role in the development of the disease, i.e., FC [22].

The *hexA* gene is involved in the production of the bacterium's capsule, which helps the bacteria avoid phagocytosis. Similarly, the *hyaD/hyaC* gene is responsible for the biosynthesis of the bacterium lipopolysaccharide (LPS) outer core and the production of hyaluronic acid capsules (HACs) [23]. This enables *P. multocida* to evade the chicken's immune response, resist phagocytosis, and colonize the host cells. The *hyaD/hyaC* gene also allows *P. multocida*'*s* to have a heme uptake system, enabling the bacterium to utilize heme as an iron source and scavenge iron from the host's tissues [22, 23]. These mechanisms contribute to *P. multocida*'*s* ability to overcome the host defenses, enhancing its virulence, and pathogenicity, ultimately establishing FC in chickens [24].

### 2.4. Transmission

The transmission of FC is significantly facilitated highly by infectious chicken and wild birds, which serve as carriers for *P. multocida* throughout their lives. The *P. multocida* takes 5–8 days to fully develop [25] and is then excreted in bird feces and oronasal discharge, where they can persist for up to 3–4 weeks in water and for 4 months in the soil [26]. Thus, the disease can be spread among the flock through direct contact. Similarly, by indirect contact with infectious droppings, contaminated feed ingestion, inhaled, and carrier bird secretions, *P. multocida* can also be transmitted and cause the disease [27]. There have been no instances of the direct or indirect transmission of FC from chickens to people or vice versa due to the varied susceptibilities of the species [25].

### 2.5. Epidemiology

Although FC is prevalent worldwide, the epidemiology of the disease in Ethiopian reveals a high prevalence across various regions, particularly in central areas like Oromia [28]. The occurrence of FC is common during the rainy season due to the multiple stressors present at this time, including high temperature and humidity, concurrent infection, and malnutrition [14]. In addition, it occurs in moist environments, which increases the organism's survival time in the environment, as well as in water sources, and insects, for several months [29]. Due to *P. multocida's* capacity to persist asymptomatically in carrier birds after clinical indications disappear, the bacterium can cause frequent outbreaks in chicken production [30]. This highlights that FC significantly contributes to overall poultry mortality rates in Ethiopia, suggesting that it is one of the main infectious diseases alongside Newcastle disease and Salmonellosis [10].

### 2.6. Clinical Signs

**Figure 1:**
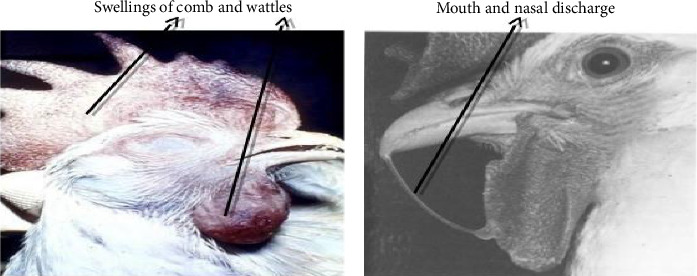
Acute form of chicken FC case. The chicken is presented with swelling associated with the blacking of combs and wattle (a) and the dropping of oronasal discharge (b) [11].

In the clinical sign of FC, the disease can exist in either acute or chronic form in affected chickens [12]. For example, in the acute case, the chicken can die before showing any signs. Also, the chicken might be presented with backward retracting of the head, ruffled feathers, off-feed and water, and dull, yellowish to gray diarrhea that progresses to watery-greenish if untreated early. Moreover, their respiratory rate is increased, and they may exhibit dyspnea, coughing, fever, and depression. Similarly, the disease can also present with oral mucous on the beak, nasal discharge, facial edema blackening of the comb, and wattles (Figure 1) [11].

**Figure 2:**
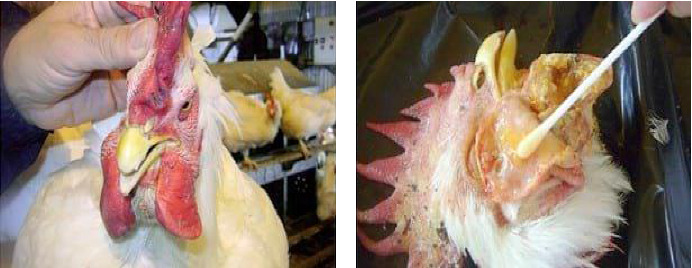
Chronic form of FC case. Chronic cases of FC with different clinical signs, such as extreme enlargement of the comb and wattle (a) and the leakage of purulent fluid (yellow caseum material) from the wattle incisions (b) [11].

On the other hand, the chronic form of FC in chickens can be asymptomatic even if the disease is present. However, following an acute form, FC spreads chronically to the localized area and is associated with clinical signs such as respiratory rales, coughing, and oronasal discharge [10]. Moreover, the disease FC in chickens can be characterized by local inflammation and lesions in the respiratory tract, conjunctiva, and nearby head tissues, which are frequently suppurative [12]. Swelling on the comb and wattles, in the legs and foot pad, as well as hock joints, can cause lameness. This could increase the number of chickens dying from FC, especially those on farms because of the high levels of contact and suffocation present in poultry (Figure 2) [11].

### 2.7. Diagnosis of FC

The diagnosis of FC can be somewhat more complicated when it presents with other bacterial diseases. For example, infectious coryza, salmonellosis, colibacillosis, and listeriosis in chickens, as well as erysipelas and chlamydiosis in turkeys, can cause clinical symptoms and lesions that resemble that of FC [31]. Therefore, the art of diagnosing FC involves both physical and clinical examination, as well as laboratory analyses. These analyses include pathological findings from affected chickens and bacteriological examination of the causative agent, *P. multocida* from suspected chicken cases [32, 33].

#### 2.7.1. Physical and Clinical Examination

The diagnosis of FC in chickens using this method is associated with finding and demonstrating various symptoms and clinical signs indicative of the disease's presence. These finding include acute mortality, with sudden deaths, fever, cyanosis (bluish discoloration of the skin, wattle, and comb), ruffled feathers, oronasal discharge, and loss of appetite. Investigating signs of greenish watery or mucoid diarrhea, depression, labored breathing, and, in some cases, finding lack of coordination or convulsions before death can also suggest the occurence of FC in chicken [10–12].

#### 2.7.2. Laboratory Analyses

The diagnosis and differentiation of FC under laboratory protocols involves the bacteriological examination of the causative agent, *P. multocida*, from various clinical samples' chickens consistent with the disease. This technique also includes investigating typical diagnostic clinical signs or lesions and microscopically observing bacteria that show bipolar staining in smears of different tissues, such as the liver, lung, and heart [29]. Moreover, the use of different molecular techniques, along with serological assays, aids in the diagnosis of FC in chickens [34].

##### 2.7.2.1. Conventional Method

Even though conventional methods are not as sensitive or fast as molecular techniques, they are still used to isolate and identify *P. multocida* based on its cultural morphology, Gram-staining properties, and biochemical characteristics [35]. During a cultural examination of a suspected case of FC, representative samples need to be grow on blood agar and chocolate agar. However, prior culturing, these media need to be supplemented with 5% defibrinated sheep blood to support the growth of *P. multocida* [29].

On the other hand, tryptone soya agar supplemented with 10% horse serum is also commonly used in vitro cultures of *P. multocida,* in addition to Mueller–Hinton agar (MHA) and brain heart infusion (BHI). Similarly, due to inhibitors, *P. multocida* does not grow on MacConkey agar and is thus used to distinguish it from other Gram-negative bacteria [36]. According to the Gram-staining pattern, being small Gram-negative, and having cocco-bacilli with bipolar staining characters that often occur single, in pairs, and occasionally in chains through a microscope suggests the bacterium to be *P. multocida*. Moreover, as indicated in Table 1, different biochemical and sugar fermentation tests are applied to isolate and identify *P. multocida* [35].

##### 2.7.2.2. Molecular Technique

Serologically, the diagnosis of *P. multocida* strain is not commonly used because it requires high-quality antisera, which is difficult to prepare and implement for clinical use [37]. Therefore, molecular sequence typing methods have been highly recommended to overcome the drawbacks of serological and conventional methods. This is due to their reliance on bacterial genetic material through specific primers, as well as the evaluation of emerging novel infections and early detection of causative agents for infection with accuracy as well reproducibility [18].

Capsular and LPS genotyping, as well as MLST, and/or virulence genotyping, are the most frequently used molecular typing methods for *P. multocida* strain differentiation [38, 39]. Among these, the capsular genotyping method, applying highly specific primers, was developed to detect different bacterial serogroups of the *cap* gene. These include *hyaD/hyaC* for A, *bcbD* for B, *dcbF* for D, *ecbJ* for E, and *fcbD* for F serogroup [40]. Similarly, molecular detection of FC involves the use of Nucleic Acid Amplification Tests (NAATs) and Matrix-Assisted Laser Desorption/Ionization-Time of Flight (MALDI-TOF) Mass Spectrometry through PCR assays. MALDI-TOF can be used as an alternative to 16S rRNA sequencing for the identification of *P. multocida* and other many bacterial strains [41]. PCR has also been used to identify and confirm *P. multocida* infections from chickens' clinical samples [42].

### 2.8. Treatment

When the chickens present with FC symptoms, they need to receive one antibiotic treatment or a combination of more antibiotics. Antibiotics such as sulfonamides, tetracycline, neomycin, quinolones (norfloxacin), sulfamerazine, tylosin, erythromycin, sulfamethazine, streptomycin, florfenicol, and penicillin are the choices for treating FC in suspected chicken cases. These antibiotics are administered for five consecutive days in the water supply or feed [25].

### 2.9. Multidrug Resistance (MDR) of *P. multocida*

The MDR in *P. multocida*, the pathogen responsible for FC in chickens, is a growing concern in Ethiopia and other regions [43]. Recent studies have highlighted alarming trends in antibiotics resistance among *P. multocida* isolates, complicating treatment options and raising public health concerns [18].

For example, a study conducted on isolates from chickens in Ethiopia revealed that a substantial percentage of *P. multocida* strains were resistant to commonly used antibiotics such as tetracycline, amoxicillin, and entrofloxacin [44]. This resistance is particularly alarming as it limits the effectiveness of standard therapeutic interventions aimed at controlling FC outbreaks [45].

The emergence of MDR strains is often attributed to the over use and misuse of antibiotics in poultry farming practices [18]. In Ethiopia, as in many other countries, antibiotics are frequently administered not only for therapeutic purpose but also for growth promotors, leading to selective pressure that favors resistant bacterial population [46]. A study found that 25.86% of *P. multocida* isolates from pigs were resistant to three or more classes of antibiotics, indicating a worrying trend that could extend to poultry if similar practices continue.

Moreover, the spread of antibiotic resistance genes among bacterial populations poses a risk not only to animal health but also to public health due to the potential transmission of resistant pathogens through the food chain [45, 47]. Thus, continuous surveillance and responsible antibiotic stewardship are essential to mitigate the impact of MDR *P. multocida* in Ethiopian poultry and ensure effective treatment options remain available. Implementing strict regulations on antibiotic use and promoting alternative management strategies can help address this pressing issue.

### 2.10. Control and Prevention

Understanding the transmission of FC in chickens is crucial for developing effective control measures due to its enormous impact on the poultry industry [11]. Knowing biosecurity and preventing rodents, wild birds, cats, and dogs from accessing their feces within coops and outdoor enclosures are the best strategies to prevent FC. All diseased chickens should be promptly removed if found among the flock and disposed of by burying or burning, along with their carcass, to prevent scavengers from accessing them for consumption. Never introduce new birds to the flock because they could be carriers of the diseases. Preventing stress as much as possible and maintaining a high level of hygiene is also important [29].

On the other hand, efforts to control FC in Ethiopia include vaccination programs utilizing both live attenuated and inactivated vaccines. These vaccines can be delivered through water and feed supplies and should be implemented as preventative measures to lower the prevalence of the disease [25]. For example, the formalin-inactivated FC vaccine (FI-FC) is the most commonly used vaccine in the country's vaccination program. However, its effectiveness is limited because it provides only short-term protection and does not adequately induce mucosal immunity [29].

To address this limitation, Ethiopia is demonstrating its commitment to advancing poultry health and productivity by adopting and innovating new vaccine technologies, even as countries worldwide work to control FC through vaccination. Among Ethiopia's new vaccine technologies, the gamma-irradiated mucosal vaccine is a novel formulation that has shown reliable and promising results in clinical trials. This vaccine, administered intranasally or intraocularly, demonstrated a remarkable 100% protection rate in vaccinated chickens when challenged with virulent strains of *P. multocida*, compared with 80% protection conferred by the traditionally FI-FC vaccine [48]. Thus, utilizing this effective vaccine to track the spread and impact of FC among chicken flocks is highly significant, in addition to improving the chickens' management system and ensuring biosecurity practices.

### 2.11. Risk Factors of the Disease

The course of FC may be affected based on various risk factors associated with the disease occurrence. These include host factors (age, breed, species, and management system), bacterial strains, previous antibiotics application, and vaccination, as well as environmental factors. Being stressed, immunocompromised, and having poor management systems are among the host factors that can determine the morbidity and mortality of chickens [21]. These conditions enable *P. multocida* to cause the disease, as it lives as commensal in the respiratory tracts of many avian species [49].

Furthermore, older or laying flocks are more susceptible and commonly affected by FC than younger ones due to aging [50]. The severity of the disease increases when *P. multocida* is coinfected with other respiratory pathogens [51]. This shows that there is no single bacterial virulence feature mechanism that has been found to correlate with observed disease occurrence, although environmental and host factors seem to influence the onset and severity outcomes [20].

### 2.12. Status of FC in Ethiopia

FC affects both domestic and wild birds worldwide, leading to high morbidity and mortality rates [52]. It is particularly sever in chickens, turkeys, and waterfowl, with outbreaks reported across various regions including Africa, Asia, and Americans [53]. Another study conducted in Bangladesh by Saha in 2021 also reported the presence and prevalence of FC, with 38.6% detection rate through phenotypic and genotypic characterization. This indicates a high circulation of *P. multocida* causing the disease in laying chickens in the country [43].

Since FC is prevalent worldwide, it is also a significant concern in Ethiopia's chicken production, highlighting the extent to which chicken mortality rates are affected by the disease. For example, in a recent study conducted in 2019 in the country, FC showed a high prevalence of 68% among other infectious poultry diseases, posing a threat to poultry health and productivity [28]. Chaka also reported a 65% prevalence of FC from apparently healthy chickens in various districts/zones of eastern Shewa zone, Ethiopia [54]. In addition, Asfaw reported an FC prevalence of 27.5% across different Ethiopian regions from 2018 to 2019, suggesting the occurrence of the disease in the country [10]. Due to this, Ethiopia is hindered from adequately generating income from chicken production.

The distribution, mortality rate, and morbidity of FC in the country are more associated with an ineffective disease prevention and control strategy, unimproved chicken's genetic potential, a poor management system, and a lack of veterinary services and necessary infrastructure [21]. To prevent the threat to the chicken population, different vaccines have proven to offer significant protection when used in disease control as Ethiopia has made tremendous progress in vaccine development and evaluation against FC [29]. Furthermore, FC remains a serious problem in Ethiopian poultry production, highlighting the necessity for further research work on treatment efforts to manage and prevent the disease, following disease diagnosis as there are insufficient published studies in Ethiopia.

## 3. Conclusion and Recommendations

FC is a prevalent bacterial disease affecting the upper and lower respiratory tract of many chicken populations. It is caused by *P. multocida*, a highly contagious Gram-negative bacterium. FC is transmitted among healthy flocks either directly or indirectly from infected, or carrier birds, resulting in significant financial losses across various chicken sectors. Once the disease occurs in the chicken, it can manifest as either acute or chronic, with different clinical signs based on the form of the disease. This review address the current trends in the diagnosing of FC in suspected chicken case, using both clinical examination and laboratory-based bacteriological analyses, such as capsular and lipopolysaccharide genotyping, as well as PCR assays. It also highlights the antibiogram patterns of *P. multocida*, which help track the spread of the disease among the chicken population in the study area. Therefore, to better understand the burden of FC in chickens, further studies should be conducted to determine the occurrence or prevalence of the disease. This will help design and improve prevention and control measures, including robust vaccination programs against FC in the city. In addition, it is important to educate and raise awareness among poultry owners about critical biosecurity measures to prevent disease and spread within flocks.

## Figures and Tables

**Table 1:** Biochemical and sugar fermentation profile of *P. multocida.*

Biochemical tests	*P. multocida*	Sugar fermentation	*P. multocida*
Oxidase	+	Lactose	−
Catalase	+	Sucrose	+
Indole production	+	Glucose	+, but no gas
Urease activity	−	Trehalose	V
Citrate utilization	−	Arabinose	V
Methyl red	−	Maltose	−
Voges Proskauer	−	Mannitol	v/+
Nitrate reduction	−	Mannose	+

*Note:* Most strains positive (+), most strains negative (−), variable reactions (v).

## Data Availability

The data used to support this review have been included within the article.
